# Supplementary material for: Gut microbiota modulates lung fibrosis severity following acute lung injury in mice
Source: Commun Biol. 2022 Dec 22;5:1401. doi: 10.1038/s42003-022-04357-x (PMC9772329; doi:10.1038/s42003-022-04357-x)
Supplement: Supplementary file 2 — Supplemental Information [file 42003_2022_4357_MOESM2_ESM.pdf]

Supplementary Figures

Gut microbiota modulates lung fibrosis severity following acute lung injury in mice

Ozioma S. Chioma[1], Elizabeth K. Mallott, Austin Chapman[1], Joseph C. Van Amburg[1], Hongmei Wu[1], Binal Shah-Gandhi[1], Nandita Dey[1], Marina E. Kirkland[1], M. Blanca Piazuelo[5]; Joyce Johnson[4], Gordon R. Bernard[6], Sobha R. Bodduluri[7], Steven Davison[8], Bodduluri Haribabu [7], Seth R. Bordenstein[2, 3, 4], Wonder P. Drake*[1, 4]

Supplemental Fig. 1: Housing conditions does not impact morbidity and mortality during saline treatment.

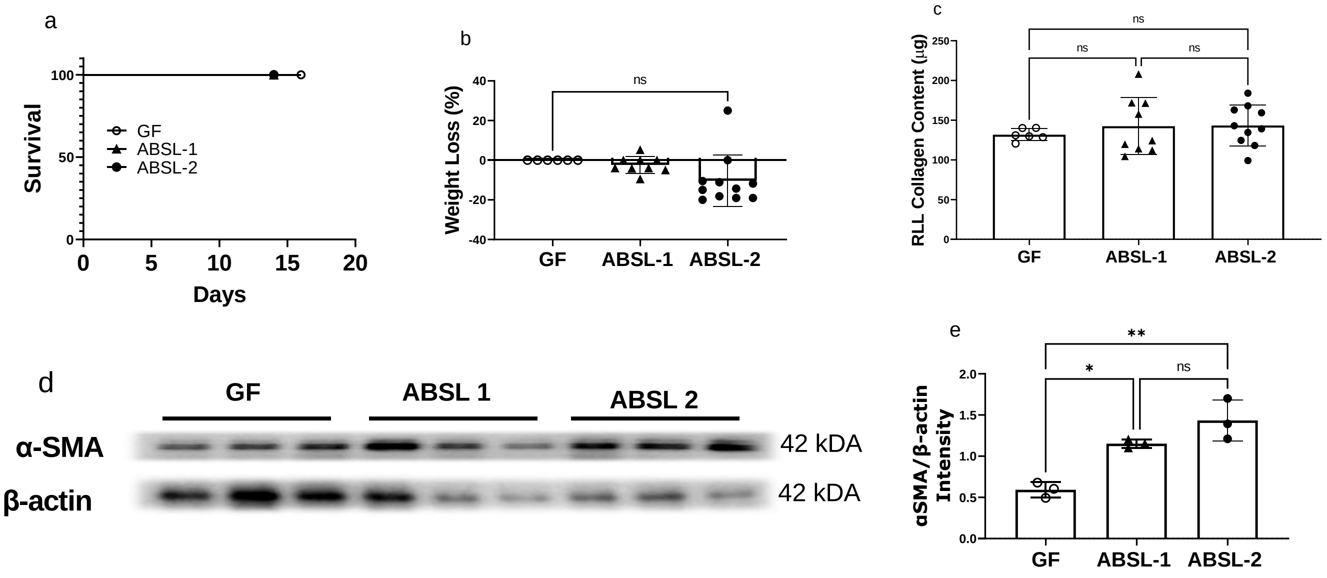

(a) Survival curve of mice housed in 3 separate facilities and intranasally inoculated with saline. **(b)** Weight loss of mice in the saline treatment cohorts; **(c)** Sircol assay of mice housed in 3 separate facilities and treated with saline (control group); **(d)** immunoblot of mice lung samples with antibodies against alpha smooth muscle actin and beta actin in bleomycin treated cohort; **(e)** quantification of immunoblot analysis; Data are reported as mean ± SD with each dot representing an individual mouse. Statistical significance was assessed using a one-way ANOVA with Sidak's multiple comparison's test for b, c; subpanel e was analyzed using Bonferroni's test due to an n=3 for each group. *P<0.05 **P < 0.01, ****P< 0.0001; ns = no significance; RLL: right lower lobe. n= 3-11 mice

Supplementary Fig. 2: Species evenness contributes to alpha diversity in ABSL-1 housed mice.

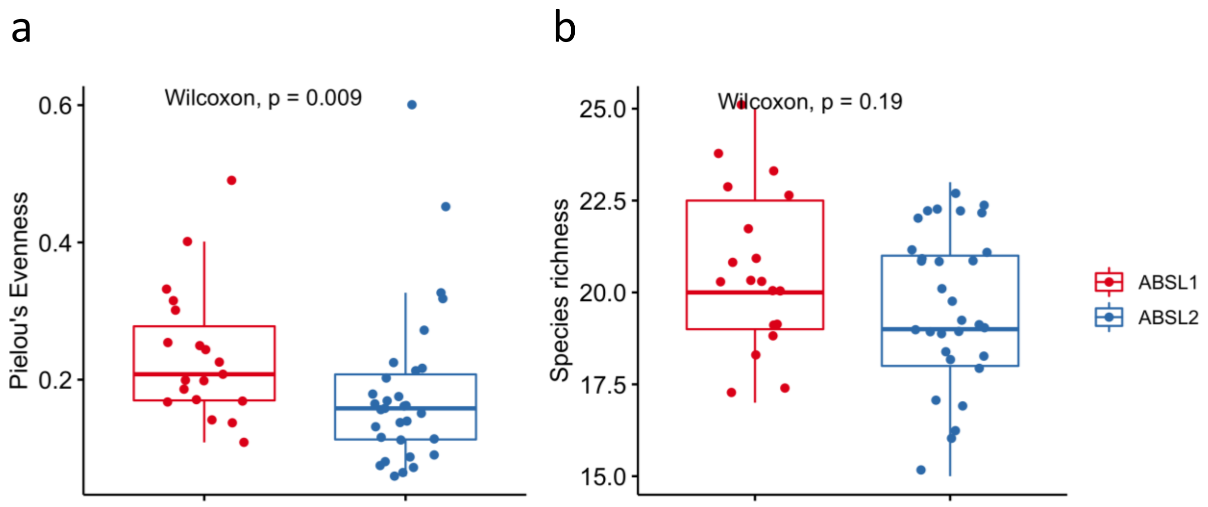

(a) Pielou's evenness and **(b)** species richness were assessed in ABSL-1 and ABSL-2 reared mice. Each dot represents an individual mouse, and median and interquartile range are indicated in the figure.

Supplemental Fig. 3: Nonlinear multidimensional scaling (MDS) plot showing differences in microbial taxonomic composition.

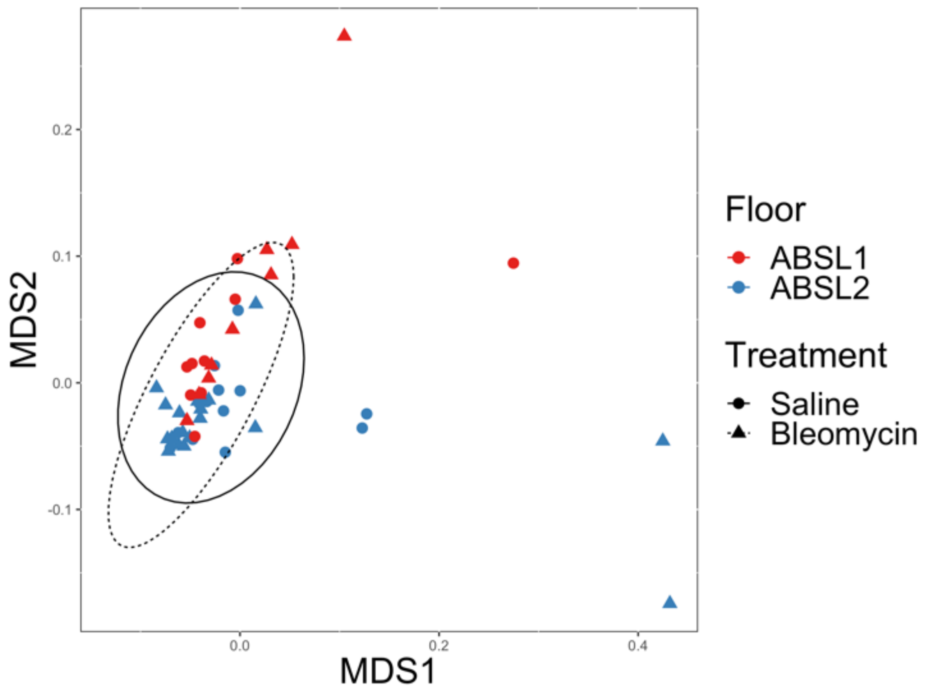

Nonlinear multidimensional scaling (MDS) plot showing differences in microbial taxonomic composition of the gut microbiome based on Bray-Curtis index in ABSL1 or ABSL2 mice). n = 13-27 mice per cohort. The experiment was independently conducted once.

Supplemental Fig. 4: Functional profiles are not different between floor or treatment when using Bray-Curtis dissimilarity matrices.

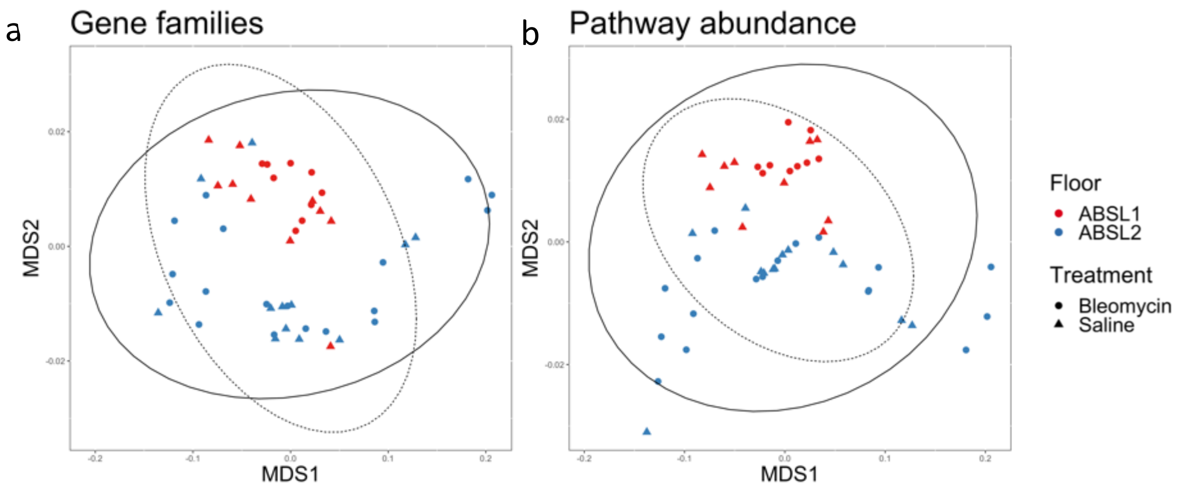

(a) Gene family abundance and **(b)** MetaCyc reaction pathway abundance profiles based on Bray-Curtis dissimilarity matrices.

Supplementary Fig. 5a-d: Representative histogram depicting IL-6 expression on CD4+ T cells in GF, ABSL-1 and ABSL-2 bleomycin treated mice.

a. $CD4^{+}$ IL-6 $^{+}$ T Cells (%)

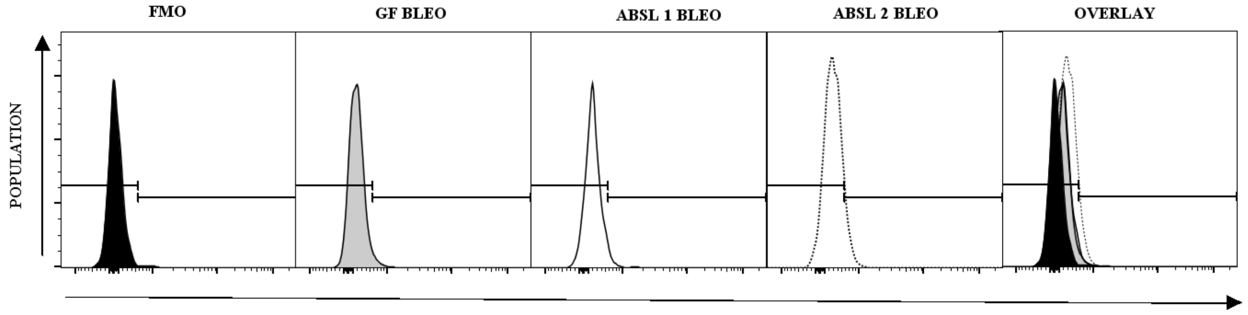

b. $CD4^{+}$ GP130 $^{+}$ T Cells (%)

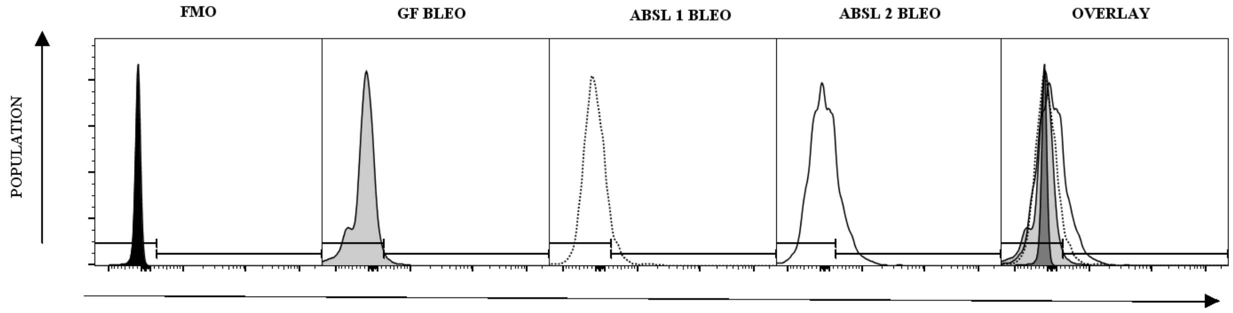

c. $CD4^{+}$ PD-1 $^{+}$IL-17A^{+} T Cells (%)

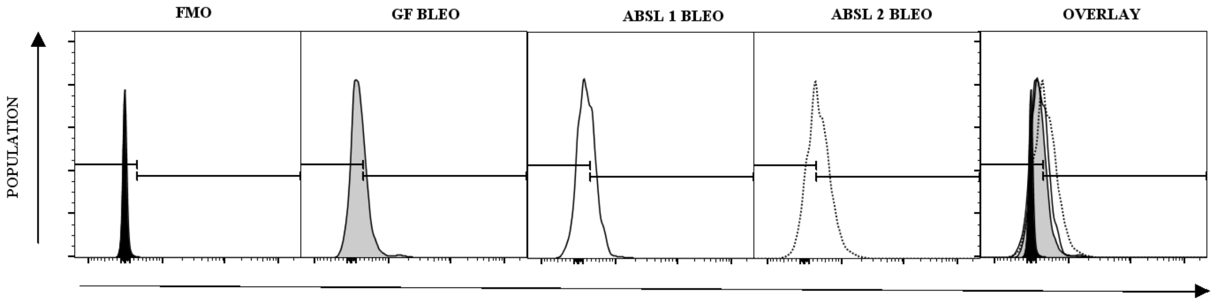

d. $CD4^{+}$ pSTAT3 $^{+}$ T Cells (%)

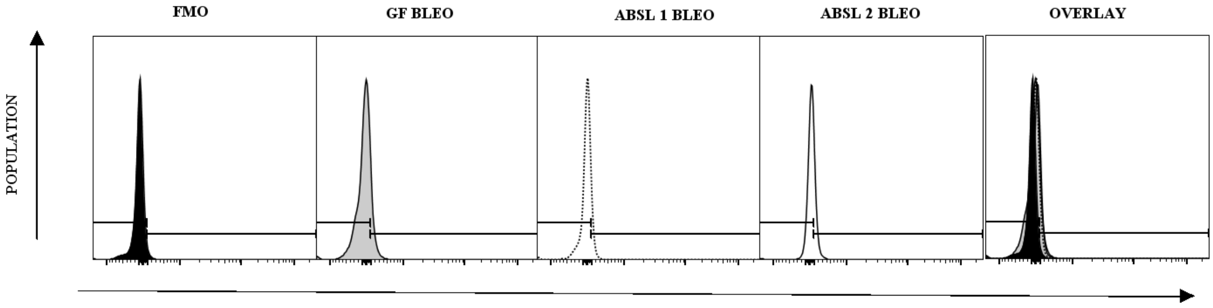

Representative histogram depicting **(a)** IL-6A+, **(b)** GP130, **(c)** PD-1+ IL-17A +, and **(d)** pSTAT3 expression on CD4+ T cells in GF mice, mice housed in ABSL 1 and ABSL2 Facilities. GF: Germ-free, ABSL-1: Animal Biosafety Level 1, ABSL-2: Animal Biosafety Level 2

Supplemental Fig. 6: Gut microbial diversity and taxa associate with inflammatory pulmonary lung responses.

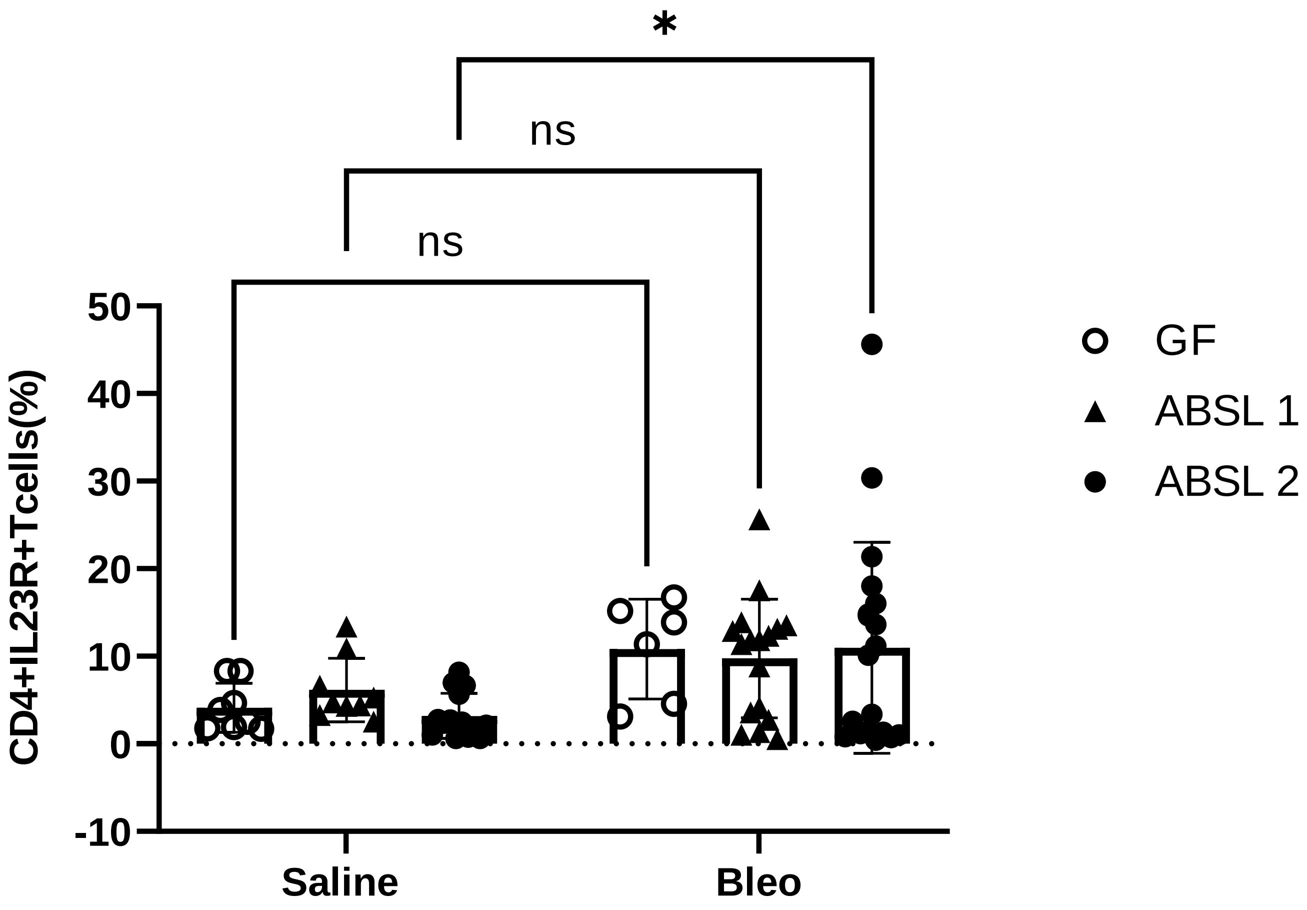

Single cell lung suspensions were obtained from mice housed in three different facilities (GF, ABSL-1 and ABSL-2). Flow cytometry analysis was conducted for IL-23R expression on CD4+T cells. Statistical significance was assessed using a two-way ANOVA with Tukey's multiple comparison's test. *$P<0.05$, **$P < 0.01$, ****$P< 0.0001$, ns = no significance n=6-19.

Supplemental Fig. 7: Western Blot image of Collagen-1 alpha (COL1A)

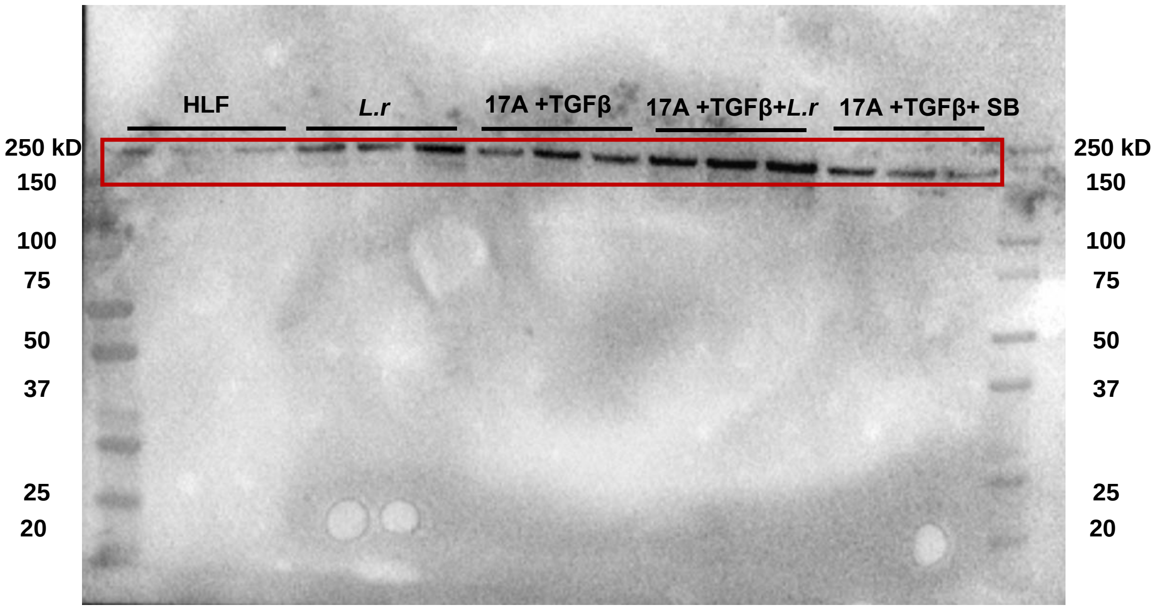

Immunoblot of Human Lung Fibroblast (HLF) assayed for collagen type 1A (col1a) production after HLF's are treated with rIL-17A and TGFβ1, supernatant from Lactobacillus grown to log phase and rIL-17A + TGFβ1 and sodium butyrate. Collagen-1 alpha (COL1A) shown at 210 kDA

Supplemental Fig. 8: Western Blot image of GAPDH

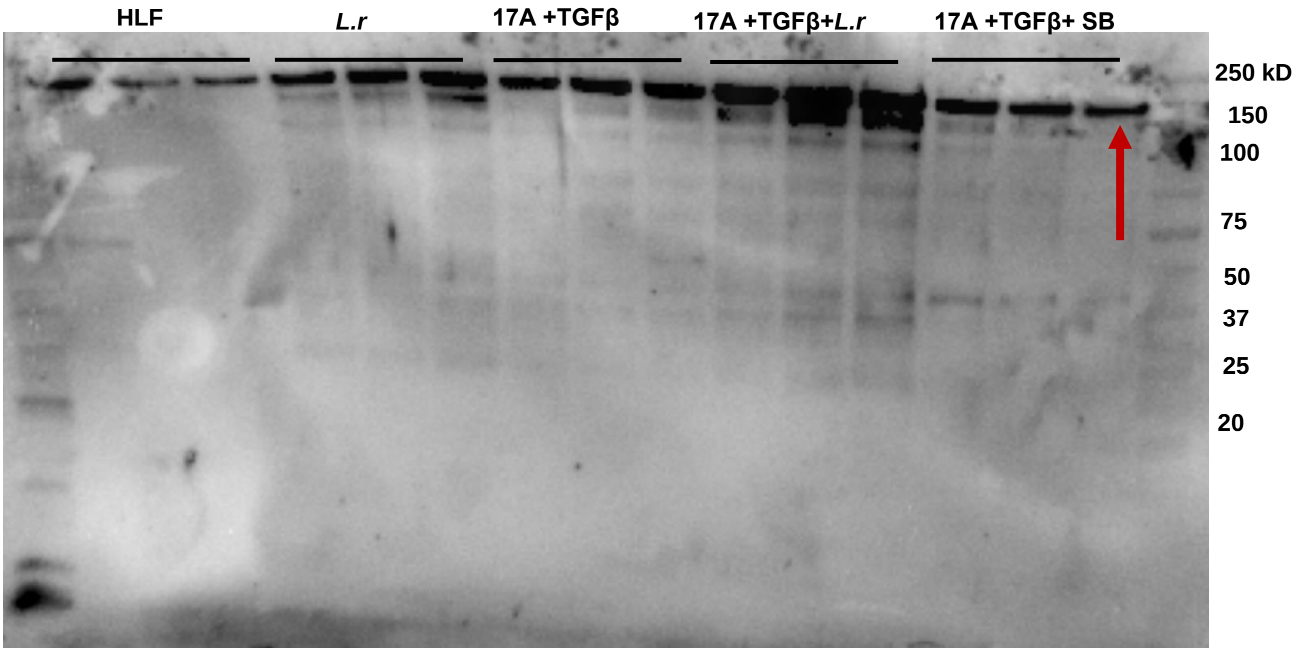

Immunoblot of Human Lung Fibroblast (HLF) assayed for GAPDH production after HLF's are treated with rIL-17A and TGFβ1, supernatant from *Lactobacillus rhamnosus* grown to log phase and rIL-17A + TGFβ1 and sodium butyrate. GAPDH Tetramer shown at 146 kDA.

Supplemental Table 1: Antibodies used for flow cytometric analysis of murine single cell suspensions.

Antibody	Vendor Catalog No	Host organism; antibody type	Clone	Conc (μg)/volume (μL) per test*
CD3 Alexa Fluor 700	Biolegend 100216	Rat Monoclonal	17A2	0.25 μg/μL
CD4 APC-Cy7	Biolegend 100414	Mouse Monoclonal	GK1.5	0.1 μg/μL
IL-6 APC	Biolegend 504808	Rat Monoclonal	MP5-20F3	0.04 μg/μL
GP-130 PE	Biolegend 149404	Rat Monoclonal	4H1B35	0.02 μg/μL
PD-1 PE-Cy7	Biolegend 135216	Rat Monoclonal	29F.1A12	0.1 μg/μL
IL-17A PE	Biolegend 506904	Rat Monoclonal	TCII-18H10.1	0.1 μg/μL
STAT3 (pY703) Pacific Blue	BD 560312	Mouse Monoclonal	4/P-STAT3	10 μg/μL
IL-23R APC	Biolegend 150906	Rat Monoclonal	12B2B64	0.02 μg/μL
*Test = 1 million cells in 50μl of staining buffer				

Supplemental Table 2: Microbiome comparison of parent and gavaged murine fecal pellets.

Group 1	Group 2	Bray-Curtis distance (mean±stddev)	Jaccard distance (mean±stddev)
Stool of ABSL1 mice	Stool of ABSL2 mice	0.158±0.116	0.301±0.088
Stool of germ-free mice gavaged with ABSL1 stool	Stool of ABSL1 mice	0.980±0.039	0.866±0.138
Stool of germ-free mice gavaged with ABSL2 stool	Stool of ABSL2 mice	0.942±0.101	0.739±0.144
Stool of germ-free mice gavaged with ABSL1 stool	Stool of germ-free mice gavaged with ABSL2 stool	0.890±0.136	0.772±0.239
Stool of germ-free mice gavaged with ABSL1 stool	Stool of ABSL2 mice	0.982±0.033	0.861±0.143
Stool of germ-free mice gavaged with ABSL2 stool	Stool of ABSL1 mice	0.965±0.056	0.755±0.139

Average Bray-Curtis dissimilarities and Jaccard distances between ABSL1 stool, stool from ABSL1-gavaged germ-free mice, ABSL2 stool, and stool from ABSL2-gavaged germ-free mice.
