# Supplementary material for: Gut microbiota modulates lung fibrosis severity following acute lung injury in mice
Source: Commun Biol. 2022 Dec 22;5:1401. doi: 10.1038/s42003-022-04357-x (PMC9772329; doi:10.1038/s42003-022-04357-x)
Supplement: Supplementary file 5 — Reporting Summary [file 42003_2022_4357_MOESM5_ESM.pdf]

Corresponding author(s): Wonder Drake MD

Last updated by author(s): 12/06/2022

Reporting Summary

Nature Portfolio wishes to improve the reproducibility of the work that we publish. This form provides structure for consistency and transparency in reporting. For further information on Nature Portfolio policies, see our Editorial Policies and the Editorial Policy Checklist.

Statistics

For all statistical analyses, confirm that the following items are present in the figure legend, table legend, main text, or Methods section.

n/a	Confirmed	
☐	☒	The exact sample size (n) for each experimental group/condition, given as a discrete number and unit of measurement
☐	☒	A statement on whether measurements were taken from distinct samples or whether the same sample was measured repeatedly
☐	☒	The statistical test(s) used AND whether they are one- or two-sided<br>*Only common tests should be described solely by name; describe more complex techniques in the Methods section.*
☐	☒	A description of all covariates tested
☐	☒	A description of any assumptions or corrections, such as tests of normality and adjustment for multiple comparisons
☐	☒	A full description of the statistical parameters including central tendency (e.g. means) or other basic estimates (e.g. regression coefficient) AND variation (e.g. standard deviation) or associated estimates of uncertainty (e.g. confidence intervals)
☒	☐	For null hypothesis testing, the test statistic (e.g. F, t, r) with confidence intervals, effect sizes, degrees of freedom and P value noted<br>*Give P values as exact values whenever suitable.*
☒	☐	For Bayesian analysis, information on the choice of priors and Markov chain Monte Carlo settings
☒	☐	For hierarchical and complex designs, identification of the appropriate level for tests and full reporting of outcomes
☒	☐	Estimates of effect sizes (e.g. Cohen's d, Pearson's r), indicating how they were calculated

Our web collection on statistics for biologists contains articles on many of the points above.

Software and code

Policy information about availability of computer code

Data collection	Quality control analysis was conducted on the genomic DNA samples. Uniquely dual indexed libraries were prepared using the Nextera DNA Flex Kit. Whole genome sequencing was performed at PE150 on the Novaseq 6000, targeting an average of 1-2M reads per sample. Data was delivered as multiplexed FASTQ.
Data analysis	Code for all analyses can be found at github.com/emallott/PulmonaryFibrosisMicrobiota.

For manuscripts utilizing custom algorithms or software that are central to the research but not yet described in published literature, software must be made available to editors and reviewers. We strongly encourage code deposition in a community repository (e.g. GitHub). See the Nature Portfolio guidelines for submitting code & software for further information.

Data

Policy information about availability of data

All manuscripts must include a data availability statement. This statement should provide the following information, where applicable:

- Accession codes, unique identifiers, or web links for publicly available datasets
- A description of any restrictions on data availability
- For clinical datasets or third party data, please ensure that the statement adheres to our policy

All data generated or analyzed during this study are included in this published article (Supplementary Data 1 file). BioPRoject ID PRJNA899808

Human research participants

Policy information about studies involving human research participants and Sex and Gender in Research.

Reporting on sex and gender	N/A
Population characteristics	N/A
Recruitment	N/A
Ethics oversight	N/A

Note that full information on the approval of the study protocol must also be provided in the manuscript.

Field-specific reporting

Please select the one below that is the best fit for your research. If you are not sure, read the appropriate sections before making your selection.

☒ Life sciences ☐ Behavioural & social sciences ☐ Ecological, evolutionary & environmental sciences

For a reference copy of the document with all sections, see nature.com/documents/nr-reporting-summary-flat.pdf

Life sciences study design

All studies must disclose on these points even when the disclosure is negative.

Sample size	6- to 8-week-old C57BL/6J male and female mice from Jackson Labs weighing approximately 17-22g were used. 4-29 mice were used in each group
Data exclusions	Mice that did not survive the experiments were noted in the survival graphs, however data from those mice were excluded from the flow cytometry analysis
Replication	Experiments were replicated at least twice
Randomization	Each experimental group contained mice of the same genetic background and same number of male and female mice
Blinding	Blinding was done only for the Ashcroft Scoring.

Reporting for specific materials, systems and methods

We require information from authors about some types of materials, experimental systems and methods used in many studies. Here, indicate whether each material, system or method listed is relevant to your study. If you are not sure if a list item applies to your research, read the appropriate section before selecting a response.

Materials & experimental systems

n/a	Involved in the study	
☒	☐	Antibodies
☐	☒	Eukaryotic cell lines
☒	☐	Palaeontology and archaeology
☐	☒	Animals and other organisms
☒	☐	Clinical data
☒	☐	Dual use research of concern

Methods

n/a	Involved in the study	
☒	☐	ChIP-seq
☐	☒	Flow cytometry
☒	☐	MRI-based neuroimaging

Eukaryotic cell lines

Policy information about cell lines and Sex and Gender in Research

Cell line source(s)	Four Human Lung Fibroblasts (HLFs) cell lines were obtained as a kind gift from Dr. Alexander Gelbard Department of Otolaryngology - Head & Neck Surgery Vanderbilt University Medical Center. The fifth cell line was purchased from Sigma Aldrich #506-05A (The HLF Demographics are provided in Table 2). Cell lines were derived form female donors.

Authentication | The cell lines donated by Dr Gelbard were not authenticated.

Mycoplasma contamination | The lines were not tested for mycoplasma contamination.

Commonly misidentified lines (See ICLAC register) | N/A

Animals and other research organisms

Policy information about studies involving animals; ARRIVE guidelines recommended for reporting animal research, and Sex and Gender in Research

Laboratory animals	6- to 8-week-old C57BL/6J male and female mice from Jackson Labs weighing approximately 17-22g were used.
Wild animals	N/A
Reporting on sex	Findings apply to male and female mice
Field-collected samples	N/A
Ethics oversight	All murine procedures were performed according to the protocol approved by the Institutional Animal Care and Use Committee at Vanderbilt University Medical Center (protocol #M1700043; ABSL-1 and ABSL-2 facilities, and FMT experiments) or University of Louisville (protocol #20786; Germ-free facilities).

Note that full information on the approval of the study protocol must also be provided in the manuscript.

Flow Cytometry

Plots

Confirm that:

☒ The axis labels state the marker and fluorochrome used (e.g. CD4-FITC).

☒ The axis scales are clearly visible. Include numbers along axes only for bottom left plot of group (a 'group' is an analysis of identical markers).

☒ All plots are contour plots with outliers or pseudocolor plots.

☒ A numerical value for number of cells or percentage (with statistics) is provided.

Methodology

Sample preparation	All flow cytometry experiments were acquired with a 4 laser fortessa cytometer(BD Biosciences), and information on all antibodies used in this study is listed in Supplemental Table 1.
Instrument	4 laser Fortessa
Software	Data analysis was performed using FlowJo software (Tree Star, Ashland, OR).
Cell population abundance	A minimum of 50,000 events were acquired per sample.
Gating strategy	Cells were gated on singlets, live CD3+ and CD4+ cells. Live cells were gated based on forward and side scatter properties, and surface staining of cells was performed as previously described

☒ Tick this box to confirm that a figure exemplifying the gating strategy is provided in the Supplementary Information.
